# The characteristics and operations of “online pharmacies” investigated in relation to medicines popularised during the coronavirus pandemic: a cross-sectional study

**DOI:** 10.3389/fphar.2024.1346604

**Published:** 2024-02-20

**Authors:** Oria Jason Oriakhi, Hamzeh Almomani, Nilesh Patel, Parastou Donyai

**Affiliations:** ^1^ School of Pharmacy, University of Reading, Reading, United Kingdom; ^2^ School of Pharmacy, University of Jordan, Amman, Jordan; ^3^ Department of Pharmacy, Faculty of Life Science & Medicine, King’s College London, London, United Kingdom

**Keywords:** online pharmacies, internet, fake medicines, patient safety, COVID-19 pandemic, prescription-only medicines, online consultation

## Abstract

**Aim:** To explore the characteristics and operations of online pharmacies in relation to medications that gained widespread popularity and increased demand during the COVID-19 pandemic. Furthermore, to assess and compare practices between legitimate and “rogue” online pharmacies.

**Methods:** Fifteen COVID-19-pandemic-related medications were investigated through a UK-based online search. We systematically assessed the credibility of 116 retrieved online pharmacies using the factors: operational location, range of medicines sold, prescription requirements for Prescription Only Medicines (POMs), information exchange, payment/delivery, user-friendliness, legitimacy. Descriptive analysis was conducted, and legitimacy status (legitimate vs. illegitimate/rogue) was tested against relevant safety indicators using a chi-square test.

**Results:** Out of 116 “online pharmacies,” 55 (47%) were confirmed as “rogue,” 47 (41%) were verified as legitimate by the General Pharmaceutical Council (GPhC), and 14 (12%) were verified by other bodies but not by the GPhC. A total of 93 declared their “apparent” physical location of operation on the webpage of which 63 (67.7%, *n* = 93) declared a location that did not match their actual location according to the online pharmacy’s server (through their IP address). All 15 medicines analysed were readily available for purchase. A high percentage of online pharmacies offered POMs to the public (93.1%, *n* = 116). Only 23 out of the 116 online pharmacies assessed required a prescription for providing POMs, with only four of these verified as legitimate by the GPhC register, while most of the legitimate pharmacies (44 out of 47) offered online consultations as an alternative option instead of a prescription. Controlled medicines were offered by 28 online pharmacies 27 of which were deemed as rogue. Rogue online pharmacies were significantly more likely to guarantee refunds for medication, not require prescriptions for POMs, and not require an online consultation to obtain POMs.

**Discussion:** Findings reveal easy access to rogue online pharmacies, posing patient safety risks. We also found legitimate online pharmacies often offer online consultations without requiring prescriptions for POMs, raising concerns about inadequate safety checks. This emphasises the need for improved regulations for both types of online pharmacies, especially during public health crises.

## 1 Introduction

The internet is now a key method for purchasing medications, and there is a growing reliance on online pharmacies for obtaining medications (Abanmy, 2017). A study published in 2022 uncovered a remarkable ten-fold increase from 2018 to 2020 in online medicine purchases in the Hungarian population (Fittler et al., 2018; Fittler et al., 2022). The purchase of medicines using the internet has also gained significant attention in the news media, highlighted by a recent study (Almomani et al., 2023b), that emphasised a growing public concern surrounding online medicine purchases.

An online pharmacy refers to internet retailers that sell non-prescription and prescription medicines directly to patients through the mail (Dineen-Griffin and Usman, 2021). While online pharmacies offer advantages such as convenience, privacy, and cost comparison, they also pose significant risks to the public if they are not operating legally; the risks include poor medication quality, safety concerns, financial risks, availability of fake medicines, and a lack of accountability (Gray, 2011; Abanmy, 2017; Jain et al., 2017; Lobuteva et al., 2022; Almomani et al., 2023a; Almomani et al., 2023c).

The internet hosts both legitimate and illegitimate (a term often used interchangeably with the term “rogue”) online pharmacies. Legitimate online pharmacies are those that have gained accreditation from official regulatory bodies. For example, online pharmacies in the UK must be registered with the General Pharmaceutical Council (GPhC) in order to operate legally (GPhC, 2023). The GPhC is the regulatory body overseeing all pharmacies in the UK. It maintains a comprehensive list of registered pharmacies, accessible to the public through their website, which serves as a vital resource for verifying the legitimacy of online pharmacy operations (GPhC, 2023). Rogue online pharmacies, unlike licensed and regulated counterparts, operate without proper authorisation from regulatory bodies, potentially endangering the public by facilitating unregulated online sales of medicines (Fittler et al., 2013). In fact, a stricter definition of unregulated online pharmacies might be “online seller of medicines” as technically speaking in the UK, descriptions such as “pharmacy” are restricted to registered pharmacies only (UK government, 2018).

A common practice of rogue online pharmacies is engaging in unlawful and unsafe activity, such as selling prescription medicines without a prescription, defrauding consumers, and selling fake, illegal, or unapproved medicines (LegitScript, 2021; Ozawa et al., 2022; Almomani et al., 2023a; Limbu and Huhmann, 2023a). There are normally warning signs indicating that an online pharmacy is rogue such as offering prescription medication without a prior consultation, or the lack of certification, licensing, or regulatory details on the website (The Independent Pharmacy, 2021), however, presently owners of these rogue pharmacies make a great deal of effort to conceal their identities and appear legitimate to deceive the public. Due to this misrepresentation, there have been concerns about patient safety which became more prevalent during the coronavirus (COVID-19) pandemic as the internet became an extremely popular way to purchase medication (Jairoun et al., 2021). Furthermore, the allure of bulk discounts and price reductions offered by rogue online pharmacies raise concerns about the potential exploitation of individuals seeking medicines during the COVID-19 pandemic (Ozawa et al., 2022).

The uncertainties resulting from the COVID-19 pandemic arguably also increased the people’s health anxieties, which in turn, influenced consumer behaviours of purchasing medicines from the internet (Dubey et al., 2020; Fittler et al., 2021; Almomani et al., 2023a). As the public searched for COVID-19 cures, public figures and influencers with huge social powers advertised the use of unlicensed or unproven medicines (without proven clinical evidence against COVID-19) causing significant demands for such medications during the pandemic (Vaduganathan et al., 2020; Almomani et al., 2023a). Furthermore, people were observed bulk purchasing medications as a way of protecting themselves against the unknown impact of the pandemic (Almomani et al., 2023a). Due to public hysteria sparked by COVID-19, the number of medicines dispensed from online pharmacies increased. In England, for instance, this number rose by 45% in 2020, increasing from 29 million in 2019 to 42 million (Wickware, 2021). The rise in the number of consumers purchasing medicines online, along with associated risks of this type of purchases, underscores the need to investigate the characteristics and operations of online pharmacies.

A study published in 2013 demonstrated the ease with which patients could access rogue online pharmacies (Fittler et al., 2013). The study involved an internet search to identify online pharmacies selling specific medicines in the United States. Out of 136 online pharmacy websites monitored over 4 years, a high prevalence of rogue pharmacies was found, accessible through the widely used search engine, Google. This type of study helps highlight the ongoing challenges that regulators face in regulating the online pharmacy space, however, the 2013 study did not consider the impact of the COVID-19 pandemic as it was conducted more than 10 years ago. Thus, the data/information collated is likely outdated due to the rapid growth in the use of online pharmacies during the COVID-19 pandemic.

Our study aims to explore characteristics and operations of online pharmacies, specifically focusing on the accessibility of medicines that garnered attention during the COVD-19 pandemic in the UK. Our study seeks to assess the ease of public access to these medicines using the internet, and whether online pharmacies adhere to necessary procedures before selling them. This will be done by assessing easily-accessed online pharmacies against characteristics deemed relevant to the legitimacy and credibility of an online pharmacy such as identification of the operator; types of medicines sold; prescription requirement; payment and delivery; user friendliness and appearance; and legitimacy verification. This study is crucial for public safety, as it sheds light on the prevalence and risks associated with online pharmacy practices during the COVID-19 pandemic. By identifying issues such as accessibility to specific medicines and adherence to necessary procedures, our findings empower consumers to make informed choices. Regulatory authorities can use our study to strengthen or create regulations and their enforcement, ensuring the authenticity and safety of online-sold medications.

## 2 Methodology

### 2.1 Selection of medicines

The selection of medications for this study was informed by the impact of COVID-19 on medication sales and dispensing rates. Each active pharmaceutical ingredient (API) investigated was chosen based on its popularity during the COVID-19 pandemic and the significant increase in interest, prescribing, or purchasing compared to the pre-pandemic period. A total of 15 APIs were selected, and Supplementary Appendix S1 provides details on the included APIs along with the rationale for their selection.

The selected APIs are classified under three UK legal categories, of general sales list (GSL), where their sale does not necessitate supervision by doctors or pharmacists, Pharmacy medicines (P medicines), requiring pharmacist supervision for their sale, and prescription-only medicines (POMs), requiring authorisation and supervision from a licensed prescriber through a prescription (MHRA, 2022). These classifications adhere to the legal categories outlined in the Human Medicines Regulations 2012 in the UK (UK Government, 2012).

### 2.2 Search strategy

Using the Google Chrome browser and the Google search engine, a systematic internet search was conducted from the UK with the objective of identifying the online pharmacies that were selling one or more of the 15 selected APIs chosen for this study. In line with the methodology of Fittler et al. (2013), the identification of online pharmacies was done by utilising the search terms “buy,” “online,” and “pharmacy” in combination with the active pharmaceutical ingredients (APIs) (for example, “buy Ivermectin,” “Ivermectin online,” “Ivermectin pharmacy”). Both the generic and most frequently used brand name of the API were used. The search was repeated for all 15 selected APIs, and the first 20 valid results that appeared, including the paid advertisement results, were included in the analysis. According to one study, 75% of the population normally do not go past the first page of search results (Ryan, 2021), thus documenting the first 20 valid search results would cover more than 75% of online pharmacies and websites the public may visit if they conducted a similar search. Webpages that failed to load, were not in English or could not be translated, and those from which medication could not be purchased were all excluded from the documented search results. The search strategy was conducted by (OJ) and verified by (PD). The search process was conducted by (OJ) and cross-checked by (HA). Critical review of the final manuscript was provided by (NP).

Using this method, a total of 300 records were collected, documented, and examined during the period between November 2021 and December 2021. These records were then narrowed down to 116 after eliminating duplicate online pharmacies (Supplementary Appendix S2). The process of selecting the 116 pharmacies involved a thorough refinement of the dataset. Following the exploration of the first 20 valid searches for each API, the dataset was systematically reviewed to eliminate duplicate online pharmacies. As an illustrative example, the online pharmacy “UK Meds” was encountered and chosen in 13 across all 15 API searches.

### 2.3 Website evaluation tools for the assessment of online pharmacies

To investigate characteristics and operations of the online pharmacies, as well as the public’s accessibility to specific medications, we utilised specific website evaluation tools for each of the included online pharmacies. These tools were adopted from the study conducted by Fittler et al. (2013). Table 1 illustrates these tools. It is important to highlight that aliases were created as part of this study with to simulate patient attempts in obtain the 15 selected medicines. However, instead of making an actual purchase of the medicines, the aliases were used to enable data collection on website characteristics and operations. Each online pharmacy’s characteristics were evaluated, summarised, and documented in a descriptive manner in an Excel spreadsheet (Supplementary Appendix S2) with the process for doing so described here.

**TABLE 1:** Website characteristics evaluation tools.

Question category	Documented characteristics of online pharmacies	Variable
Identification of the operator	Domain name	URL
	Year website was published	Date (Year)
	Declared physical location of operation	World region & country & city
	Telephone contact	Telephone number
	Location of the server according to IP address	World region & country
	Does location match IP address	Yes/No
Medicines sold (Range of medicines provided to the public)	Variety of medicines sold	Brand/Generic/Both
	Classification of medicines sold	GSL/P/POM/Veterinary*
	Number of selected medicines able to be acquired from webpage	Number/List of medicines
Prescription requirement	Requirement of prior medical prescriptions for POMs	Yes/No
Information exchange	Availability of online consultation	Yes/No
	Requirement of patient’s medical information	Yes/No
	Availability of general product information	Detailed/Incomplete/Not available
	Availability of the patient information leaflet	Detailed/Incomplete/Not available
Payment and delivery	Available payment methods (e.g., credit card, money transfer, PayPal)	Number of methods
	Delivery time	Days
	Cost of delivery	Pounds (sterling)/US dollars
	Refund policy	Refund guarantee/No refund
User friendliness and appearance (subjective elements)	User-friendly navigation	Easy to use/Average/Difficult to navigate
	General appearance and design	Excellent/Average/Poor
	Customer feedback on website	Displayed/Not available
Legitimacy verification	Legitimacy according to GPhC database	Legitimate/illegitimate
	Approved by other regulatory bodies (if not regulated by GPhC)	Name of other foreign regulatory bodies/Unapproved

#### 2.3.1 Identification of the operator

In the process of identifying the operator of each online pharmacy, crucial data were collected. Every webpage was assessed by inspecting the operator’s home page and delving into the contact information; the copyright information; the “About Us” section; the FAQ (frequently asked questions) the help; and the legal (Privacy Policy and the Terms and Conditions) sectors of the webpage for data on the identification of the operator.

To determine the actual establishment year of each webpage, a combination of methods was employed, including Google searches, inspecting the webpage’s source code, and carbon dating of the webpage (Stanton, 2021). To confirm the accuracy of the declared location of operation on online pharmacy webpages, we assessed the actual location by determining the server’s location through the IP address. The IP address was obtained using the Command Prompt on the Windows 8 program, and then it was entered into IP Lookup programs (such as Iplocation net, 2021; Barber, 2021) to determine the precise location of the server.

#### 2.3.2 Range of medicines provided to the public

The range of medication offered to the public was analysed as it is a factor that affects patient safety and can be a crucial way to entice the public into purchasing medications from the webpage (Gray, 2011; Barber, 2021). The variety of medicines sold (branded/generic), the classification of the medicines sold (GSL/P/POMs/Veterinary), and number of selected APIs offered (from the 15 selected APIs) were assessed by scrutinizing the webpage and investigating the medication that was offered and can be acquired from the webpage. It is important to note that the categorisation of POMs is based on the classification system used in the United Kingdom, where the same API may be classified as a POMs in the UK and an OTC medication in another country. This study adopts the UK’s classification system for the purpose of analysis.

#### 2.3.3 Requirement of prescription for POMs

Due to the pandemic, some patients may have ordered POMs online from different countries to obtain their desired medicines without a prescription, however, the law had not changed, and prescriptions are required for POMs. Thus, every webpage was assessed to investigate the prescription requirements for POMs.

Recognising the important role of prescriptions in ensuring patient safety and adherence to regulatory standards, the investigation aimed to check whether online pharmacies imposed appropriate prescription requirements for POMs. This assessment involved exploring the webpage to determine the specific prescription protocols for obtaining POMs (either a paper prescription or an electronic prescription), shedding light on the adherence of online pharmacies to safety and legal standards in the sale of these regulated medications.

#### 2.3.4 Medication health exchange between patient and medical health provider

Medical health exchange is important in the context of individuals obtaining their medications, especially certain POMs, without prescriptions. Clear communication between an individual and healthcare provider is essential as it helps the healthcare provider to properly assess and diagnose a condition, as well as ensuring that the care they give is truly “patient-centred” and is tailored to the individual (Al Zoubi et al., 2021; Ammassari et al., 2021; Pyzik et al., 2021). Some online pharmacies may offer prescriber services where an individual completes an online consultation (online questionnaire, phone call, video call, online chat, or email) and then if the answers are adequate a prescription is then provided and sent to the pharmacy for dispensing (NHS, 2021).

This study investigated the exchange of medication-related information between a would-be patient and online pharmacy by examining several factors. These included assessing the presence of online consultations, the requirement for patients’ medical information, and the availability of general product information and patient information leaflets. The evaluation process involved interacting with medications on the webpage, specifically POMs, to ascertain whether online consultations were offered and if the online pharmacy mandated the exchange of medical information during the medication ordering process. Additionally, the study assessed the quality and quantity of general product information and patient information leaflets by inspecting the offered medications. The categorisation of these information sources was based on their quality and quantity, with designations of “detailed,” “incomplete,” or “not available,” following criteria established by Fittler et al. (2013).

#### 2.3.5 Payment and delivery

As more individuals turned to online pharmacies during the pandemic to obtain medication (Jairoun et al., 2021; Fittler et al., 2022; Almomani et al., 2023a), another important feature of their experience was the payment options on offer to them. Buying anything online requires trustworthy methods of payment and delivery, ensuring a secure and reliable transaction process. In this study, payment methods, delivery time, delivery cost, and refund policies for each online pharmacy were also assessed. This involved scrutinizing the webpage’s return policy and the delivery and shipping sections, along with a practical exploration of purchasing medicines to determine the available payment options. Data regarding the delivery time was gathered according to working days. Data regarding the delivery cost were documented in the currency the webpage was using (sterling pound, dollars, euros ….). Results for the return policy were categorised into 2 categories: refund guaranteed and no refund (unless faulty, damaged or incorrect).

#### 2.3.6 User-friendliness and appearance

Additional variables of importance were deemed to be the website’s user-friendly navigation, website appearance, and customer feedback on the website, as these elements are part of the initial interaction of the public with the online pharmacy. We evaluated the user-friendliness of the navigation of each online pharmacy and categorised this into one of three groups: “easy to use” for websites that were straightforward and easy to navigate; “average” designated for websites that had encountered minor issues, such as occasional slow loading times or slight organisation challenges; and “difficult to navigate” for websites that presented significant problems, making interaction and navigation notably challenging. General appearance and design were also categorised into 3 different categories: “excellent” if the webpage was clean and smooth with all parts of the interface placed appropriately around the webpage; “average” if the webpage looked decent with minimal problems however with not all parts of the interface placed appropriately; and “poor” if the design of the webpage looked messy causing problems for the user and with different parts of the interface placed in unsuitable places around the webpage. Customer feedback on the sites was investigated and was assessed to either have customer feedback displayed on the webpage or unavailable.

#### 2.3.7 Legitimacy according to the GPhC verification database

Categorisation of the pharmacies as legitimate or “rogue” involved verifying the legitimacy of the selected online pharmacies, as follows and included two key steps. First, the legitimacy of each online pharmacy was evaluated by entering the details of each online pharmacy, as found on their webpage, into the GPhC register (GPhC, 2023). This step ensured that the online pharmacy was listed on the GPhC register, thus confirming its legitimacy. The second step involved checking for accreditations from other regulatory bodies, especially when online pharmacies operate legally in foreign jurisdictions. In such cases, they may be primarily regulated by the regulatory body within the specific nation’s jurisdiction. The accreditation systems considered included LegitScript, NABP (National Association of Boards of Pharmacy), CIPA (Canadian International Pharmacy Association), IPABC (International Pharmacy Association of British Columbia), and recognition by the Dutch Ministry of Health, Welfare, and Sport.

### 2.4 Relevant safety indictors in relation to the legitimacy status of online pharmacy

A series of safety indicators were defined to be; location of operation matching location of the server, refund policy for medication, POMs availability, prescription requirement for POMs, and provision of online consultations. The correlation between these safety indicators and the legitimacy of the online pharmacy was analysed using Chi-square tests. This analysis aimed to evaluate which safety indicator were associated with illegal operation.

## 3 Results

### 3.1 Identification of the operator

A total of 109 online pharmacies (94.0%, *n* = 116) displayed their telephone contact information on the webpage. Regarding the location of operation, 93 online pharmacies (80.2%, *n* = 116) declared their “apparent” physical location of operation. Table 2 shows the distribution of where online pharmacies declared their location of operation. A total of 51 online pharmacies declared the UK as their location of operation followed by the USA (13 online pharmacies). However, all the 116 online pharmacies’ actual locations of operation were able to be assessed through their IP address as shown in Table 2. A total of 67 online pharmacies’ servers were located in the USA (57.8%, *n* = 116) while 17 online pharmacies were located in the UK (14.7%, *n* = 116).

**TABLE 2:** Declared and Actual location of operation.

Location of operation	Location of operation as appeared on the website	Actual location of operation (using the IP address)
Europe	60	37
North America	18	69
Asia	9	5
Oceania	4	5
South America	1	0
Africa	1	0
Not declared	23	0

Out of the 93 online pharmacies that declared their physical location of operation, only 30 (32.3%, *n* = 93) had actual server location that matched their declared physical location of operation on the webpage (according to IP address). Potential factors contributing to this mismatch may include intentional misrepresentation, server hosting services located in a different region, or changes in server locations post-declaration. Further investigation is needed to understand the validity behind this inconsistency and its implications.

### 3.2 Range of medicines provided to the public

In sum, 97 online pharmacies offered both branded and generic medication (86.7%, *n* = 112) with 4 online pharmacies (3.6%, *n* = 112) offering exclusively branded medication and 11 online pharmacies (9.9%, *n* = 112) offering exclusively generic medication. The type of medication sold could not be assessed for 4 online pharmacies.

Regarding the range of medications available online, 108 online pharmacies offered POMs medicines (93.1%, *n* = 116), while 82 online pharmacies offered P medicines (70.7%, *n* = 116), while GSL were offered by 59 online pharmacies (50.9%, *n* = 116) and Veterinary medications offered by 7 online pharmacies (6%, n = 116). A total of 5 (4.3%, *n* = 116) online pharmacies offered all 4 categories (POMs, P, GSL and Veterinary) and 51 (44.0%, *n* = 116) offered POMs, GSL and P medicines, while 2 (1.7%, *n* = 116) offered exclusively GSL medications, and 31 (26.7%, *n* = 116) offered exclusively POMs.

Table 3 provides an overview of the online availability of the selected 15 APIs in 116 online pharmacies. The APIs available on 3 of the 116 online pharmacies could not be assessed as these online pharmacies did not provide visibility or access to their medication offerings without requiring payment, an online consultation, or a valid electronic prescription or prescription token. By examining the remaining 113 online pharmacies, the range of online pharmacies selling at least one of the API’s is 21–78, with azithromycin, diclofenac, aspirin, paracetamol and metformin being the top 5 API’s advertised/offered. Alprazolam was the least API advertised/offered. Interestingly, alprazolam was sold from 21 out of 21 (100%) online pharmacies classed as rogue. Similarly, in contrast, paracetamol was sold by the highest count of legitimate pharmacies (34/63 offering the API).

**TABLE 3:** Number of online pharmacies offering selected APIs (*n* = 113).

API selected to be assessed	Number of online pharmacies offering selected API	Number of rogue online pharmacies offering selected API
Paracetamol	63 out of 113 (55.8%)	29 (46.0%, *n* = 63)
Dapagliflozin	37 (32.7%)	29 (78.4%, *n* = 37)
Metformin	63 (55.8%)	40 (63.5%, *n* = 63)
Rosuvastatin	58 (51.3%)	35 (60.3%, *n* = 58)
Ezetimibe	48 (42.5%)	34 (70.8%, *n* = 48)
Formoterol	46 (40.7%)	24 (52.2%, *n* = 46)
Hydroxychloroquine	43 (38.1%)	32 (74.4%, *n* = 43)
Ivermectin	52 (46.0%)	35 (67.3%, *n* = 52)
Chloroquine	49 (43.4%)	31 (63.3%, *n* = 49)
Fluoxetine	48 (42.5%)	38 (79.2%, *n* = 48)
Alprazolam	21 (18.6%)	21 (100.0%, *n* = 21)
Aspirin	66 (58.4%)	39 (59.1%, *n* = 66)
Diclofenac	78 (69.0%)	39 (50.0%, *n* = 78)
Azithromycin	77 (68.1%)	41 (53.2%, *n* = 77)
Diazepam	28 (24.8%)	27 (96.4%, *n* = 28)

### 3.3 Requirement of prescription for POMs

Of the 108 online pharmacies that offered POMs to the public, 23 (21.3%, *n* = 108) requested prescriptions (either a paper prescription or an electronic prescription from a GP or another healthcare professional) before selling POMs, while 85 (78.7%, *n* = 108) did not request a prescription. For the 23 online pharmacies requesting prescriptions, 2 did not declare their location, while 21 provided location information on their webpage. Among these, the distribution by country was as follows: USA (5), UK (4), India (3), Canada (3), Australia (3), Middle East (1), Malaysia (1), and Netherlands (1).

### 3.4 Medication health exchange between patient and medical health provider

In our study, 60 online pharmacies (51.7%, *n* = 116) requested patient health information before selling medicines and provided online consultations. Among these, 56 (93.3%, *n* = 60) offered POMs. Notably, of the 108 online pharmacies selling POMs, a noteworthy 52 (48.2%, *n* = 108) did not request patient health information and did not offer any consultations.

**FIGURE 1:**
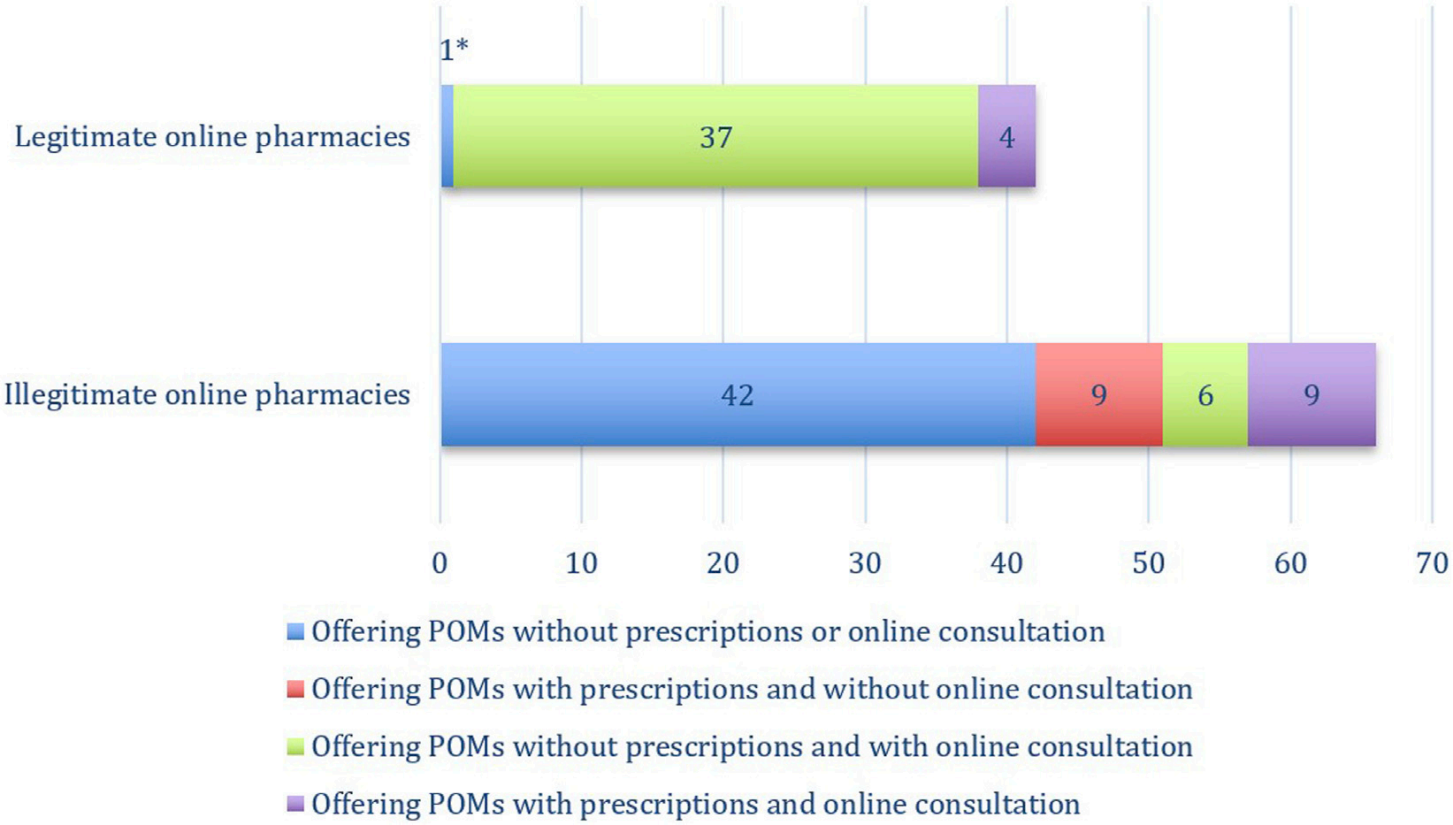
Prescription practices and consultation services in online pharmacies: A comparative analysis of legitimate and illegitimate online pharmacies. * The online pharmacy, once deemed legitimate and offering POMs without the need for pre-existing prescriptions or online consultations, has now been delisted from the GPhC. Furthermore, its website is currently unreachable, indicating potentially that regulatory measures taken by the GPhC following our initial assessment.

Figure 1 illustrates distinct patterns in the prescription practices and consultation services offered by online pharmacies. A relevant finding is that the majority of legitimate online pharmacies that offered POMs, 37 out of 42 (88.1%, *n* = 42), sold POMs with requesting prescriptions from the customers, while instead, they offered online consultation services by filling in forms that could be easily manipulated by the patient to obtain the desirable POMs.

**FIGURE 2:**
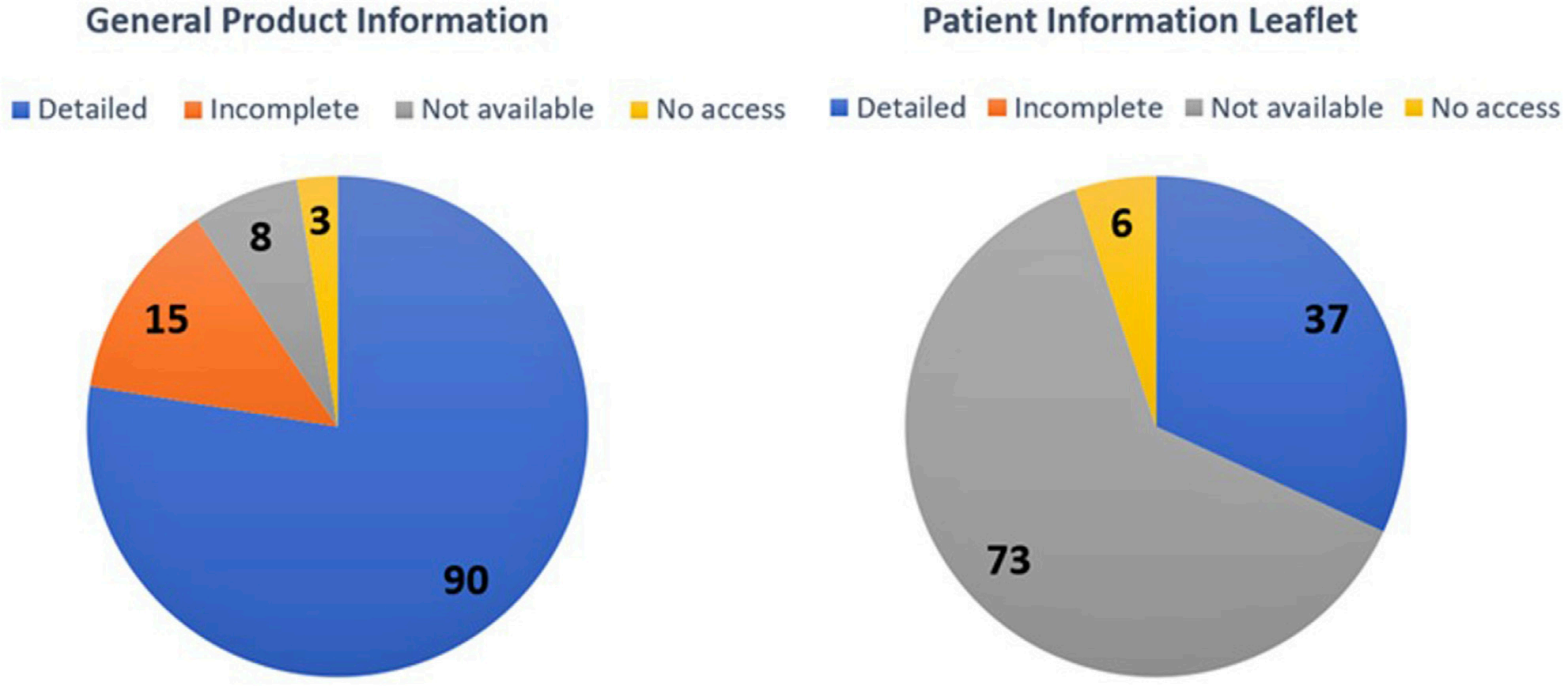
The availability and quality of general product information and patient information leaflet within the online pharmacies’ webpages.

Regarding the availability of the general product information and patient information leaflets for the medicines, Figure 2 illustrates the prevalence of online pharmacies that offer these patient information sources on their webpage and the quality of each. A sum of 90 online pharmacies offered detailed general product information (77.6%, *n* = 116), while 73 online pharmacies did not offer the patient information leaflet (62.9%, *n* = 116).

### 3.5 Payment and delivery

When evaluating the refund policy on medication for each of the 116 online pharmacies, a total of 9 online pharmacies’ refund policies could not be accessed. Out of the remaining 107 online pharmacies, 82 online pharmacies’ (76.6%, *n* = 107) refund policy stated that there is no refund on medication (unless faulty, damaged or incorrect) while 25 (23.3%, *n* = 107) guaranteed a refund for medication.

A total of 4 of the 116 online pharmacies’ payment methods were not able to be accessed. Of the remaining 112 online pharmacies, a total of 28 online pharmacies (25%, *n* = 112) offered more than 5 payment methods to pay for medicines, while 84 (75%, *n* = 112) offered 5 or fewer payment methods to complete transactions. A sum of 90 online pharmacies (87.5%, *n* = 112) used Visa or Mastercard as a payment option. Twenty online pharmacies used less conventional payment methods such as Bitcoin (17.9%, *n* = 112).

Out of 116 online pharmacies, the delivery charges for 7 online pharmacies could not be accessed. Of the remaining 109 online pharmacies, 81 offered free delivery (74.3%, *n* = 109). Considering all of the online pharmacies assessed there were at least 5 different currencies used (Pound sterling, Dollars, Euro, Naira, Rupee).

### 3.6 User-friendliness and appearance

Table 4 shows the number of online pharmacies that displayed customer feedback on the website as well as showing the quality of the online pharmacies assessed against its user-friendly navigation and general appearance and design. Of the 116 online pharmacies assessed, 76 online pharmacies were easy to use (65.5%, *n* = 116), with 64 online pharmacies having an excellent appearance (55.2%, *n* = 116), and 92 online pharmacies displaying customer feedback on their website (79.3%, *n* = 116).

**TABLE 4:** Quality of online pharmacies user friendliness and appearance (*n* = 116).

Parameter	Selected outcome parameters	Number of online pharmacies
User-friendly navigation	Easy to use	76 out of 116 (65.5%)
	Average	37 (31.9%)
	Difficult to navigate	3 (2.6%)
General appearance and design	Excellent	64 (55.2%)
	Average	49 (42.2%)
	Poor	3 (2.6%)
Customer feedback on website	Displayed	92 (79.3%)
	Not Available	24 (20.7%)

### 3.7 Legitimacy verification according to GPhC verification database

**FIGURE 3:**
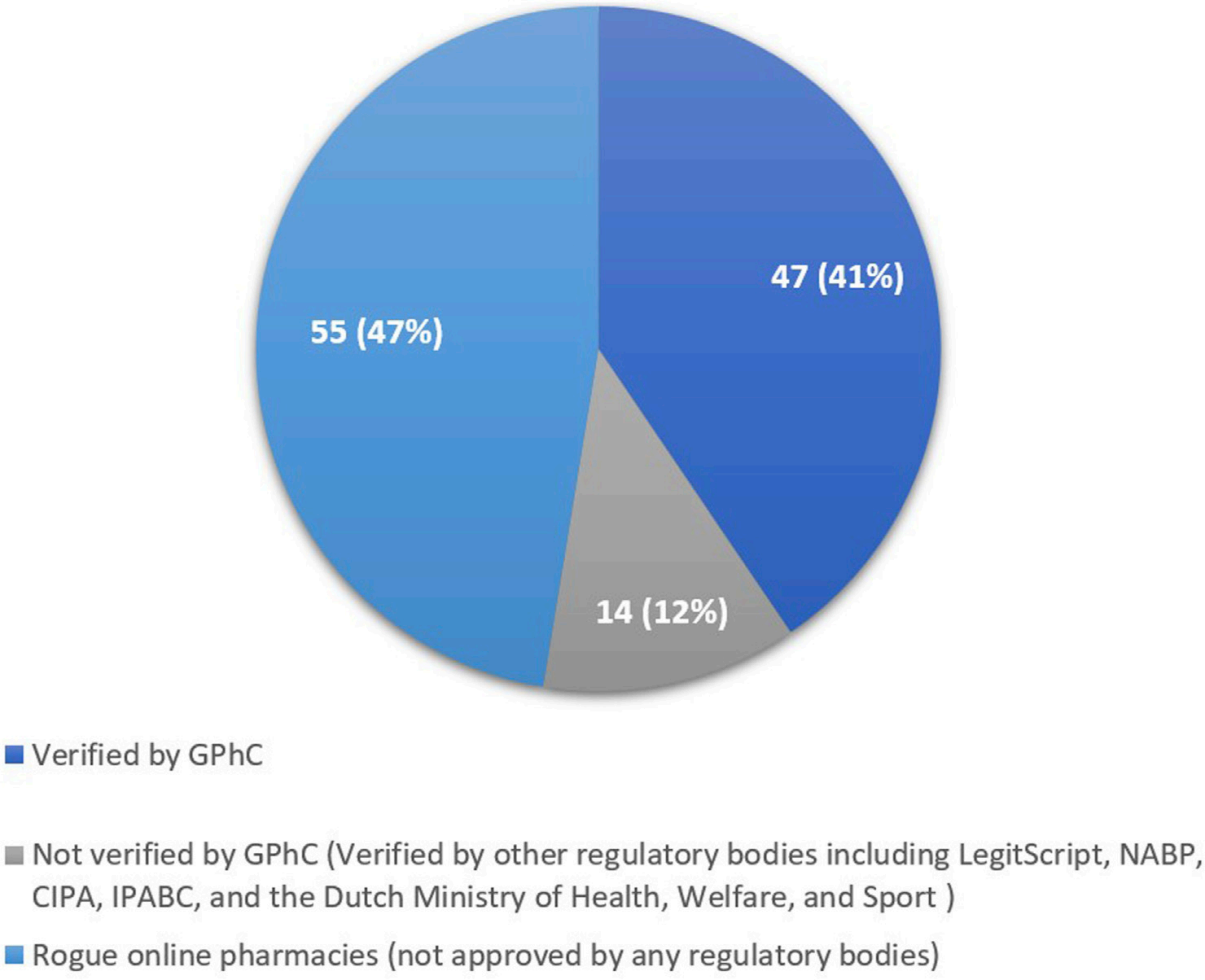
The percentage of the 116 online pharmacies categorised as “legitimate” or “rogue.”

Out of the 116 online pharmacies assessed, 47 were verified against the GPhC registers, indicating compliance with regulatory standards (Figure 3), while 14 online pharmacies had received approval from other regulatory bodies but not the GPhC, and the accrediting regulatory entity for each is detailed in Supplementary Appendix S2. In contrast, 55 were deemed rogue online pharmacies, lacking proper authorisation or failing to meet established standards. Only 4 of the 55 rogue online pharmacies declared their location of operation as the UK, while the remaining 51 operated from outside the UK.

### 3.8 Safety indicators in relation to the legitimacy status

Table 5 shows the correlation between medication safety indicators and the legitimacy of online pharmacies. Out of the 47 online pharmacies that were verified by the GPhC, 38 (80.9%, *n* = 47) did not require a prescription (for a POM transaction), 4 (8.5%, *n* = 47) require prescription, and the situation for the remaining 7 (14.9%, *n* = 47) could not be determined. Additionally, 44 of the 47 legitimate online pharmacies (93.6%, *n* = 47) provided the option of having an online consultation, while 3 online pharmacies (6.4%, *n* = 47) did not offer this option either.

**TABLE 5:** Chi-squared test results.

Selected outcome parameters	Verified by GPhC	Not verified by GPhC	Chi-squared test (*p* = 0.05)
Declared location of operation match the actual location of server (*n* = 93, 23 not applicable)			
Yes	16 (17.2%)	14 (15.1%)	Not significant
No	31 (33.3%)	32 (34.4%)	
Refund policy for medication (*n* = 107, 9 not applicable)			
Refund guaranteed	4 (3.7%)	21 (19.6%)	Significant
No refund	42 (39.3%)	40 (37.4%)	
POMs availability (*n* = 116)			
Yes	42 (36.2%)	66 (56.9%)	Not significant
No	5 (4.3%)	3 (2.6%)	
Require prescription to acquire POMs (*n* = 108, 8 not applicable)			
Yes	4 (3.7%)	19 (17.6%)	Significant
No	38 (35.2%)	47 (43.5%)	
Require online consultation to acquire POMs (*n* = 116)			
Yes	44 (37.9%)	16 (13.8%)	Significant
No	3 (2.6%)	53 (45.7%)	

Note. Totals of each parameter do not equal 116 as some information for the online pharmacies was not applicable in reference to the parameter being measured.

## 4 Discussion

This study investigates the alarming accessibility and prevalence of online pharmacies, with a substantial proportion operating outside of the controls placed. The ease of acquiring POMs without prescriptions or health checks poses significant patient safety risks, highlighting the urgency for further regulatory interventions. Notably, all the 15 medicines analysed which had gained notoriety during the COVID-19 pandemic were readily available for purchase online. This access must be weighed against their lack of proven effectiveness in preventing or treating COVID-19.

This study also reveals evidence of the online availability of controlled medicines, specifically alprazolam and diazepam, with a majority offered by “rogue” online pharmacies. Among the online pharmacies assessed, 28 offered these controlled medicines, with 27 of them classified as “rogue” online pharmacies. This ease of availability raises alarms about potential misuse or abuse due to the absence of medical oversight, aligning with a UK study that investigated the online availability of controlled medicines, revealing that 64 out of 118 examined websites provided various types of controlled medicines (Whitfield et al., 2021).

A significant revelation is the ease with which an alias, created in minutes, could be used to purchase medicines online. This exposes a major flaw in the system, lacking verification of patient identification for both legitimate and rogue websites. Additionally, the study highlights that only 23 out of 116 online pharmacies required a pre-existing prescription from the patient before dispensing POMs, with just 4 of these verified as legitimate against the GPhC register. Instead, most legitimate online pharmacies implemented online consultations or questionnaires before dispensing POMs. However, a concerning aspect is the potential for patients to manipulate responses during online consultations, obtaining desired POMs without proper safety checks. This finding aligns with concerns raised by the GPhC, emphasising unacceptable prescribing practices where medicines are prescribed based on an online questionnaire alone, with no direct interaction between the prescriber and the patient or their regular prescriber, especially in high-volume, transactional supplies of high-risk medicines (GPhC, 2022). Thus, our study emphasises the need for enhanced regulations to ensure thorough safety checks that are recommended are actually completed.

Our study reveals a concerning reality that a diverse range of POMs is readily available online without the need for a prescription. This is consistent with the findings of previous studies (Fittler et al., 2013; Boyd et al., 2017; Fittler et al., 2021; Ozawa et al., 2022; Penley et al., 2022; Sun et al., 2022) underscoring the pervasive availability of POMs without proper oversight. This finding serves as a red flag for those interested in ensuring safe medication practices and highlights an unsettling loophole for access to medications. Our findings align with prior research highlighting increased online purchases during pandemics or for otherwise hard-to-access medicines, magnifying the potential risks involved (Fittler et al., 2018; Alwhaibi et al., 2021; Jairoun et al., 2021; Moureaud et al., 2021; Almomani et al., 2023a; Almomani, 2023c). Complicating matters further is the deceptive online marketing strategies employed by rogue pharmacies as indicated by other studies (Ozawa et al., 2022; Penley et al., 2022; Limbu and Huhmann, 2023a). The enticing marketing strategies used by various online sellers of medicines, such as discounts and price reductions, create a significant patient safety concern. Our study not only contributes empirical evidence to this issue but also reinforces the imperative for regulatory interventions to ensure the safety and wellbeing of online pharmaceutical consumers.

A previous study by the authors found that people can be easily deceived by the appearance of an online seller of medicines (Almomani et al., 2023a). The assessment of user-friendliness and appearance, coupled with customer feedback, reflects the initial interactions users may have with online pharmacies. The study’s categorisation of websites based on navigation and design quality adds a valuable qualitative dimension to the evaluation. Consumer deception by the appearance of “rogue” online pharmacies was found to be a persistent issue, with most “rogue” pharmacies presenting an average or excellent appearance. The challenges in ensuring transparency and accuracy in the online pharmaceutical landscape be further hampered considering that only 32.3% of the declared and actual locations of operations aligned. A “legitimate looking” website makes it difficult for consumers to identify legitimate and safe sources for medicines from “rogue” sites, and this finding emphasises the crucial need to cultivate heightened consumer scepticism through increased awareness and education, aligning with the insights from Limbu and Huhmann (2023b). Providing consumers with information about potential risks, such as encountering rogue platforms and the prevalence of deceptive appearances, empowers them to approach the online pharmaceutical market with a careful mindset, contributing to safer online pharmaceutical markets.

Regulatory bodies in the UK have developed verification systems and guidelines for the operation of online pharmacies to improve patient safety. These are no doubt helpful for ensuring that legitimate online pharmacies understand and can operate within the rule. However, our study has shown that most of the online pharmacies available via the Google search engine were rogue and offered easy accessibility for UK consumers to high-risk POMs including controlled medicines. Further, verification seals and logos might be useless in this sense, and even misleading to consumers as potentially rogue online sellers of medicines could attempt to display fake seals or logos.

Considering the magnitude of this issue, regulatory bodies and stakeholders must explore strategies that go beyond verification systems or mere guidelines. The acceptance of cryptocurrencies like Bitcoin introduces new challenges in tracking and regulating transactions. Additionally, the prevalence of servers located in the USA suggests a global nature of online pharmacy operations.

One plausible approach is to focus on understanding consumers’ behaviours and motivations, allowing the development of targeted interventions. This could mitigate the purchasing of medicines from unsafe sources, emphasizing the need for a comprehensive, consumer-centric approach to tackle this burgeoning problem.

Another, bolder move, would be to lobby or regulate so that the search engines and providers of internet services prevent access to rogue pharmacies online altogether. The study suggests that these platforms play a pivotal role in the accessibility of rogue online pharmacies, and restricting this access could be a significant step toward safeguarding public health.

In order to strengthen the regulation and policing of websites that sell medicines in the UK, we recommend enhancing the collaboration among relevant regulatory bodies, including the MHRA, GPhC, and the Care Quality Commission (CQC). The MHRA oversees the safety and efficacy of medicines, offering pharmaceutical regulatory expertise (UK government, 2024). The GPhC, as the online pharmacy regulator, contributes insights into practice and professional standards (GPhC, 2024). The CQC, an independent regulator of health and social care services, ensures the overall quality and safety of healthcare delivery (CQC, 2024). Strengthening this collaborative effort would maximize their efforts to enhance the online pharmaceutical market safety. Initiatives such as sharing databases, information exchange, unified accreditation protocols, and a comprehensive reporting and policing system are recommended, fostering a cohesive and coordinated regulatory approach.

## 5 Limitations and future research agenda

### 5.1 Limitations

Several limitations merit consideration. First, our study’s reliance on data from only 15 APIs, while providing valuable insights, limits the generalizability of our findings to a broader range of pharmaceutical compounds. Second, as this was a cross-sectional study, the longevity of online pharmacies could not be assessed. Third, the exclusion of black market/darknet as a potential source of acquiring medication presents a limitation. Fourth, the study did not involve purchasing actual medications, relying on website characteristics for risk assessment. Finally, some website characteristics were likely inaccessible during data collection, as full verification or payment was required for certain indicators, posing constraints.

### 5.2 Future research agenda

#### 5.2.1 Online consultations assessment

Building upon our findings, future research could further explore the regulatory aspects of legitimate online pharmacies that provide consultations. For example, this could include potential risks associated with manipulating online consultation forms by patients to reach the “correct response” and obtain POMs despite a clear clinical need. This exploration could contribute to developing clearer guidelines for online pharmacies to ensure responsible and secure practices.

#### 5.2.2 Bitcoin in online pharmacies

Exploring the impact of cryptocurrency, especially Bitcoin, as a payment option in online pharmacies is a critical issue that could be explored in future research. Our study highlighted the prevalence of Bitcoin transactions in this context. Thus, prompting further investigation of the benefits, risks, and regulation around the use of cryptocurrency could contribute to informed policies and practices.

#### 5.2.3 Server location inconsistency

Building upon our findings, future research could focus on exploring the factors contributing to the mismatch between declared physical locations and actual server locations among online pharmacies. Exploring potential reasons such as intentional misrepresentation can be crucial in addressing issues related to transparency and accuracy in the virtual space.

#### 5.2.4 Exploring consumer behaviour

Future research could explore consumer behaviour in the context of online pharmaceutical purchasing. Understanding the factors influencing consumers’ decisions, motivations, and the psychological aspects behind choosing online pharmacies for purchasing medicines could provide valuable insights. While some studies have explored consumer behaviour in this context, as highlighted in a systematic review by the authors (Almomani et al., 2023c), there are gaps in research across many countries. Covering this gap could provide a more comprehensive understanding consumer behaviour and enable the development of evidence-based interventions.

#### 5.2.5 Broadening API range investigated

Future research could broaden the scope and provide a more comprehensive understanding of online pharmaceutical trends by analysing a more extensive range of APIs available online to enhance the generalizability of findings.

#### 5.2.6 Validation through medication purchases

Future studies might involve the actual purchase of medications to validate website characteristics, providing a more valid assessment of the risks associated with online pharmaceutical transactions. Analysing the content of these medicines can also help address questions around the prevalence of fake and substandard medicines.

#### 5.2.7 Longitudinal assessments

Conducting longitudinal studies and tracking online pharmacies, especially for rogue pharmacies, over time to evaluate their sustainability and adaptability could provide valuable insights into their survival strategies and tactics to evade regulators.

## 6 Conclusion

This study underscores the rampant presence of rogue online pharmacies in the UK, exploiting the demand for medicines, especially in relation to the COVID-19 pandemic. The lax adherence to necessary procedures and standards, coupled with the potential for patient safety compromise, necessitates urgent regulatory measures. With a minimal requirement for prescriptions and the potential for manipulation during online consultations, there is a crucial need for more effective regulations and policing to prevent exploitation, particularly in times of public distress like the COVID-19 pandemic. The study also suggests exploring consumer behaviours and motivations as an avenue for developing targeted interventions. A bold move is to lobby or regulate search engines and internet service providers to restrict access to rogue online pharmacies, ultimately safeguarding public health.

## Data Availability

The original contributions presented in the study are included in the article/Supplementary Material, further inquiries can be directed to the corresponding author.

## Abbreviations

MHRA, Medicines and Healthcare products Regulatory Agency; GPhC, General Pharmaceutical Council; APIs, Active pharmaceutical ingredients; GSL medicines, General sales list medicines; POMs, Prescription-only medicines; P medicines, Pharmacy medicines.
