# Supplementary material for: The characteristics and operations of “online pharmacies” investigated in relation to medicines popularised during the coronavirus pandemic: a cross-sectional study
Source: Front Pharmacol. 2024 Feb 20;15:1346604. doi: 10.3389/fphar.2024.1346604 (PMC10912318; doi:10.3389/fphar.2024.1346604)
Supplement: Supplementary file 2 [file Table1.DOCX]

**Included APIs and the rational for selecting them**

| **Classification of API** | **Selected API assessed** | **Rationale for selecting APIs** |
| --- | --- | --- |
| Non-steroidal anti-inflammatory drugs (NSAIDs) | Paracetamol, Aspirin, Diclofenac | - A study showed seven different APIs including paracetamol registered with the highest growth rate in sales and shortages over the COVID-19 outbreak (February 1st, 2020, to April 30th, 2020) when compared to the same period in the previous year (Romano et al., 2020). - Studies showed that during the pandemic about 20% of the medication bought online were non-steroidal anti-inflammatory drugs (NSAIDs) (Jairoun et al., 2021). - The first common initial symptoms of COVID-19 were fever, cough, diarrhoea, and fatigue (with fever being the clinical symptom) (Ma et al., 2021) which is why aspirin and diclofenac has been selected as two commonly used NSAIDs with anti-pyretic properties as the public will look towards these medications as treatment options. |
| Benzodiazepines | Diazepam, Alprazolam | - A study found a significant increase in the prevalence major depressive disorder and anxiety during COVID-19 pandemic (Taquet et al., 2021), while another study that the demand on controlled medicines during the COVID-19 increased (Whitfield et al., 2021). |
| Anti-glycaemic drugs | Dapagliflozin, Metformin | - A study showed seven different APIs including dapagliflozin and metformin registered with the highest growth rate in sales and shortages over the COVID-19 outbreak (February 1^st^, 2020, to April 30^th^, 2020) when compared to the same period in the previous year (Romano et al., 2020). |
| Cholesterol lowering drugs | Rosuvastatin, Ezetimibe | - A study showed seven different APIs including rosuvastatin and ezetimibe registered with the highest growth rate in sales and shortages over the COVID-19 outbreak (February 1^st^, 2020, to April 30^th^, 2020) when compared to the same period in the previous year (Romano et al., 2020). |
| Macrolide antibiotic | Azithromycin | - Furing the pandemic azithromycin had a massive increase in demand in public hospitals with an increase of 195.40% compared to pre-COVID-19 period and these demands were parallel to those demands in community pharmacies (Ammassari et al., 2020). |
| Antimalarials | Hydroxychloroquine, Chloroquine | - A study showed seven different APIs including chloroquine and hydroxychloroquine registered with the highest growth rate in sales and shortages over the COVID-19 outbreak (February 1^st^, 2020, to April 30^th^, 2020) when compared to the same period in the previous year (Romano et al., 2020). |
| Anthelmintic | Ivermectin | - A study showed a growth in demand of Ivermectin during the COVID pandemic outbreak. (Fittler et al., 2021) |
| Selective Serotonin Reuptake Inhibitor (SSRI) | Fluoxetine | - The COVID-19 pandemic has had a huge toll on people’s mental state worldwide with a significant increase of over 25% in prevalence of both major depressive disorders and anxiety disorders causing an increase in purchasing and use of antidepressants (with fluoxetine being most commonly used antidepressant) (Taquet et al., 2021). |
| Long-acting beta agonist (LABA) | Formoterol | - A study showed seven different APIs including formoterol registered with the highest growth rate in sales and shortages over the COVID-19 outbreak (February 1^st^, 2020, to April 30^th^, 2020) when compared to the same period in the previous year (Romano et al., 2020). |
